# At-Risk Phenotype of Neurofibromatose-1 Patients: A Multicentre Case-Control Study

**DOI:** 10.1186/1750-1172-6-51

**Published:** 2011-07-13

**Authors:** Emilie Sbidian, Sylvie Bastuji-Garin, Laurence Valeyrie-Allanore, Salah Ferkal, Jean P Lefaucheur, Alain Drouet, Pierre Brugière, Cédric Vialette, Patrick Combemale, Sébastien Barbarot, Pierre Wolkenstein

**Affiliations:** 1 Université Paris Est (UPEC), LIC EA4393 (Laboratoire d'Investigation Clinique), F-94010 Créteil, France; 2 Assistance Publique-Hôpital Paris (AP-HP), Hôpital Henri-Mondor, Pôle Recherche Clinique-Santé Publique, F-94010 Créteil, France; 3 Assistance Publique-Hôpital Paris (AP-HP), Hôpital Henri-Mondor, Service de Dermatologie, F-94010 Créteil, France; 4 Assistance Publique-Hôpital Paris (AP-HP), Hôpital Henri-Mondor, Centre de référence des Neurofibromatoses, F-94010 Créteil, France; 5 INSERM, Centre d'Investigation Clinique 006, F-94010 Créteil, France; 6 Assistance Publique-Hôpital Paris (AP-HP), Hôpital Henri-Mondor, Service de Physiologie-Explorations fonctionnelles, F-94010 Créteil, France; 7 UPEC, EA 4391 (Excitabilité Nerveuse et Thérapeutique), F-94010 Créteil, France; 8 Service de Neurologie, Hôpital d'Instruction des armées, Lyon, F-69000, France; 9 Assistance Publique-Hôpital Paris (AP-HP), Hôpital Henri-Mondor, Service de Neuroradiologie, Créteil, F-94010, France; 10 Assistance Publique - Hôpitaux de Paris (AP-HP), Hôpital Henri Mondor, Unité de Recherche Clinique; 11 Service de Dermatologie, Centre Léon Bérard, Lyon, F-69000, France; 12 Réseau NF-Rhône-Alpin, Centre Léon Bérard, Lyon, F-69000, France; 13 Service de Dermatologie, CHU Hôtel Dieu, Nantes, F-44200, France

## Abstract

**Objectives:**

To assess associations between subcutaneous neurofibromas (SC-NFs) and internal neurofibromas in patients with neurofibromatosis type 1 (NF-1) and to determine whether the association between SC-NFs and peripheral neuropathy was ascribable to internal neurofibromas.

**Patients and methods:**

Prospective multicentre case-control study. Between 2005 and 2008, 110 NF-1 adults having two or more SC-NFs were individually matched for age, sex and hospital with 110 controls who had no SC-NF. Patients underwent standardized MRI of the spinal cord, nerve roots and sciatic nerves and an electrophysiological study. Analyses used adjusted multinomial logistic regression (ORa) to estimate the risk of the presence of internal neurofibromas or peripheral neuropathies associated with patients presented 2 to 9 SC-NFs, at least 10 SC-NFs as compared to patients without any (referential category).

**Results:**

Cases had a mean age of 41 (± 13) years; 85 (80%) had two to nine SC-NFs and 21 (19%) at least ten SC-NFs. SC-NFs were more strongly associated with internal neurofibromas in patients with ten or more SC-NFs than in patients with fewer NF-SCs (e.g., sciatic nerve, aOR = 29.1 [8.5 to 100] vs. 4.3 [2.1 to 9.0]). The association with SC-NFs was stronger for diffuse, intradural, and > 3 cm internal neurofibromas than with other internal neurofibromas. Axonal neuropathy with slowed conduction velocities (SCV) was more strongly associated with having at least ten SC-NFs (aOR = 29.9, 5.5 to 162.3) than with having fewer SC-NFs (aOR = 4.4, 0.9 to 22.0). Bivariate analyses showed that the association between axonal neuropathy with SCV and sciatic neurofibromas was mediated by the association between SC-NFs and sciatic neurofibromas.

**Conclusion:**

The at-risk phenotype of NF-1 patients (i.e. NF-1 patients with SC-NFs) is ascribable to associations linking SC-NFs to internal neurofibromas at risk for malignant transformation and to axonal neuropathies with slowed conduction velocities. Axonal neuropathies with SCV are particularly common in patients with at least ten SC-NFs.

**Registration details:**

ORPHA86301

## Introduction

Neurofibromatosis type 1 (NF-1 [MIM 162200]) is a common autosomal dominant disorder associated with increased morbidity and mortality[1]. Neurofibromas are the hallmark of NF1. They are benign tumors that arise from connective tissue of nerve sheaths, especially the endoneurium. We used the classification proposed by Riccardi which defines four categories of neurofibromas [2]: (i) cutaneous neurofibromas, presenting as an exophytoc tumor moving with the skin on examination, (ii) subcutaneous neurofibromas that lie deeper in the skin, do not move with it, are firm to palpation and may be tender (iii) internal or deep neurofibromas may involve any nerve anywhere along its length and are not palpated. They are therefore identified later in their course of growth (iv) plexiform neurofibromas may involve multiple fascicles and branches, and extend into surrounding structures. Clinical investigation identifies thickened hypertrophic skin, hyperpigmentation of tissue and subcutaneous tumor [2,3].

Subcutaneous neurofibromas (SC-NFs) were independently associated with mortality among adults with NF-1 in two independent populations from France [4] and North America [5]. The main causes of death were spinal cord compression by internal neurofibromas and malignant peripheral nerve-sheath tumours (MPNSTs) developed from pre-existing internal neurofibromas [4,5]. A recent study demonstrated that having at least two SC-NFs was independently associated with having internal neurofibromas [6]. However, this association needs to be characterised in detail. Furthermore, a strong association between peripheral neuropathy and SC-NFs has been reported [7]. We hypothesized that this association was related to an association between SC-NFs and internal neurofibromas located along the nerve roots.

The primary objective of this prospective matched case-control study was to characterise the association between SC-NFs and internal neurofibromas in patients routinely investigated with magnetic resonance imaging (MRI), the most sensitive method for detecting internal neurofibromas and assessing their features (e.g., type, distribution, location, and size). The secondary objective was to determine whether peripheral neuropathy was associated with having internal neurofibromas along the nerve roots. Peripheral neuropathy was detected using a routine electrophysiological study.

## Patients And Methods

### Study design

This case-control study was conducted prospectively from February 2005 to December 2008 in three hospital centres in France. For a type 1 error of 0.05, 100 cases and 100 controls provided 90% power for detecting odds ratios (ORs) greater than 3 for factors having a 20% prevalence in the general population of adults with NF-1. We decided to select 110 cases and 110 controls to allow for some patients being excluded secondarily (e.g., because of contraindications to MRI). Controls were matched individually to cases on age (± 3 years), sex, and hospital. The study was approved by the Ile-de-France IX (Paris, France) ethics committee, and all cases and controls gave their informed consent.

### Cases

The cases were identified among adults with NF-1 in our NF-network database. They met diagnostic criteria for NF-1 established at the National Institutes of Health Consensus Development Conference (n = 1 099) [8]. Among patients aged 17 years or older (n = 748), we included probands and patients with sporadic disease (n = 515) who had at least two SC-NFs (n = 192), defined as palpable nodules along peripheral nerves under the skin. We did not include patients having less than two SC-NFs (n = 259), pregnant women, and patients with contraindications to MRI (e.g., cardiac pacemaker, ferromagnetic or electronically operated stapedial implant, haemostatic clip, or metallic splinters) (n = 64).

Among these 192 potential cases, 110 cases were selected at random for study inclusion.

### Controls

For each case, we looked for a control individually matched on age, sex, and centre in the NF-network database. Potential controls were probands or sporadic NF-1 patients who were at least 17 years old and had no SC-NFs and no contraindications to MRI. When we found several controls appropriate for the same case, we selected one at random. Thus, 110 controls were selected.

### Data collection

Data, including demographic information (age, sex and body mass index) and clinical features were collected during routine clinical assessments at the neurofibromatosis clinics by one dermatologist at each centre (SF, PC, SB) (Table 1). The skin lesions were described in detail: number of café-au-lait spots, cutaneous neurofibromas (0, 1, 2-9, 10-99, ≥100), and SC-NFs (0, 2-9, 10-99, ≥100); and count and location of plexiform neurofibromas defined as benign peripheral nerve-sheath tumours involving multiple nerve fascicles or branches of major peripheral nerves. Most of the plexiform neurofibromas were cutaneous or subcutaneous and were identified clinically. Other items were coded as present or absent. Orthopaedic complications (scoliosis), neurological abnormalities (headache, epilepsy, learning disabilities), and endocrinological disorders (hypertension) were also recorded.

**Table 1:** Characteristics of adults with at least two subcutaneous neurofibromas (cases) and controls

Clinical features	Cases n = 106	Controls n = 102	*p *value*
**Matching variables**			
Female gender	62 (59)	62 (61)	
Age (years)	41 (± 13)	41 (± 14)	

**Clinical variables**			
Familial case	56 (47)	56 (45)	0.76
No cutaneous neurofibromas	13 (12)	4 (4)	0.03
Plexiform neurofibromas	66 (62)	49 (48)	0.04
Large café-au-lait spots (number)	8.6 (± 4.8)	10.1 (± 10.1)	0.07
No freckles	21 (20)	12 (12)	0.11
Lisch nodules	48 (81)	45 (80)	0.89
Scoliosis	46 (43)	42 (41)	0.75
Headache	38 (36)	41 (40)	0.52
Epilepsy	2 (2)	2 (2)	0.97
Learning disabilities	58 (57)	52 (49)	0.26

*p value obtained using chi-square test or Fisher exact or non-parametric Kruskal-Wallis test as appropriate

Data are numbers (%) or means ± 1 standard deviation

#### Identification and characterisation of internal neurofibromas

All cases and controls had a standardized MRI study of the spinal cord, nerve roots, and sciatic nerve. MRI was performed using a field strength of 1.5-T. T1- and T2-weighted nonenhanced sequences (coronal plane) including short-tau inversion-recovery (STIR) sequences were acquired using a slice thickness of 4 mm with no gap. The image matrix was 512/256. Three coronal images (cervico-thoracic spine, thoraco-lumbo-sacral spine, and pelvis/thighs) were acquired to characterize the internal neurofibromas. MRI data were reviewed by a senior radiologist (PB) who was unaware of the clinical features. We recorded the presence of internal neurofibromas, their type (along the spinal cord or sciatic nerve roots), their distribution (focal or diffuse), their size (< 3 cm or ≥3 cm), and their location (intradural or extradural).

#### Electrophysiological study

All cases and controls underwent a standardized electrophysiological study to look for peripheral neuropathy. The investigation included sensory/motor nerve conduction study for the superficial/deep peroneal nerves and the sural/tibial nerves at both lower limbs and for the median and ulnar nerves at one upper limb. The electrophysiological data were reviewed by a senior physiologist (JPL) who was unaware of the clinical features. Patients were classified as having either no neuropathy (normal nerve conduction study) or an axonal distal, sensory-predominant polyneuropathy (characterized by a bilateral and symmetric reduction of sensory nerve action potential amplitude at the lower limbs with less marked abnormalities regarding motor nerve conduction parameters and upper limb results). Among axonal neuropathies, we differentiated those with and without slowed conduction velocities (SCVs). Neuropathies with and without SCVs shared the same presentation in terms of action potential amplitude reduction, but neuropathies with SCVs additionally showed moderate but diffuse decrease of motor nerve conductions, very homogeneously, without any increased temporal dispersion or conduction block. Therefore, we assumed that neuropathies with SCVs were primarily axonal because they did not present any relevant criteria in favour of a demyelinating process [9]. We also determined the presence of proximal/nerve root involvement ("radiculopathy") according to a selective alteration (absence or prolonged latency) of the F-waves and H-reflexes at the lower limbs.

### Statistical analysis

Data were double-keyboarded and analysed using STATA software version 11 (Stata Corporation, College Station, TX, USA). All tests were two-tailed and *p *values < 0.05 were considered statistically significant. Quantitative variables are reported as mean ± standard deviation (SD) and qualitative variables as number (%).

First, we compared cases and controls using univariate analysis (chi-square test or Fisher exact test as appropriate). Then, logistic regression models were used to estimate ORs adjusted for matching variables (aORs) with their 95% confidence intervals (95%CIs) for each characteristic of internal neurofibromas (spinal cord or sciatic nerve root, intra or extradural, diffuse or focal, size, and size category < 3 cm or ≥3 cm). Similarly, we estimated aORs with their 95%CIs separately for each type of peripheral neuropathy and radiculopathy. Then, to test our hypothesis that the association between SC-NFs and peripheral neuropathy was related to internal neurofibromas along the nerve roots, bivariate analyses including peripheral neuropathies and internal neurofibromas were performed. Finally, to assess whether the number of SC-NFs was associated with internal neurofibromas and/or peripheral neuropathy, cases were classified into two groups based on whether they had two to nine SC-NFs or at least ten SC-NFs. The, risk associated with each of these categories versus the controls (no SC-NFs) was estimated using adjusted multinomial logistic regression (aOR). Control patients were the referential category.

## Results

Between 2005 and 2008, 110 cases were matched for age, sex, and hospital to 110 NF-1 controls. Four cases and eight controls were excluded secondarily because their MRI scans were not interpretable. Table 1 reports the characteristics of the 106 cases and 102 controls. The cases had a mean age of 41 (± 13) years; 59% were females, 85 (80%) had two to nine SC-NFs and 21 (19%) at least ten SC-NFs. There were no significant differences between cases and controls for age and sex (matching variables) or clinical features (e.g., dermatological characteristics, orthopaedic complications, and neurological abnormalities) except the cutaneous and plexiform neurofibromas. Significantly greater proportions of cases than controls had no cutaneous neurofibromas (*p *= 0.03) and at least one plexiform neurofibroma (*p *= 0.04). Cases had fewer large café-au-lait spots compared to controls but the difference was not statistically significant (*p *= 0.07).

Table 2 summarises the results of the univariate analysis. The presence of SC-NFs was strongly associated with having paraspinal neurofibromas (aOR, 4.3; 95%CI, 2.2 to 8.2) and sciatic neurofibromas (aOR, 6.1; 95%CI, 2.9 to 13). The associations tended to be stronger for diffuse vs. focal paraspinal neurofibromas, intradural vs. extradural neurofibromas, and internal neurofibromas measuring at least 3 cm vs. less than 3 cm. Similarly, for sciatic neurofibromas, diffuse distribution and tumour size of at least 3 cm showed trends toward stronger associations with SC-NFs than focal distribution and size smaller than 3 cm, respectively. Finally, the presence of axonal neuropathy with SCVs was significantly associated with having SC-NFs (aOR, 7.7; 95%CI, 1.6 to 36.6) (Table 3).

**Table 2:** Presence of internal neurofibromas in cases and controls

	Cases, n = 106 (%)	Controls, n = 102 (%)	Odds Ratio* (95%CI)	*p *value**
**Paraspinal neurofibromas**	**54 (51)**	**20 (20)**	**4.3 (2.2 - 8.2)**	**< 10^-4^**
**Distribution**				
None	52 (49)	82 (81)	1	
Focal	26 (25)	16 (15)	2.6 (1.2 - 5.3)	
Diffuse	28 (26)	4 (4)	14.7 (3.8 - 57.3)	< 10^-4^
**Location**				
None	52 (49)	82 (81)	1	
Extradural	37 (35)	17 (16)	3.2 (1.6 - 6.4)	
Intradural	17 (16)	3 (3)	8.8 (2.3 - 33.9)	< 10^-4^
**Size**				
None	52 (49)	82 (81)	1	
< 3 cm	30 (28)	14 (14)	3.4 (1.6 - 7.2)	
≥3 cm	24 (23)	6 (5)	6.3 (2.3 - 17.4)	< 10^-4^

**Sciatic neurofibromas**	**50 (47)**	**13(13)**	**6.1 (2.9 - 13.0)**	**< 10^-4^**
**Distribution**				
None	63 (60)	90 (88)	1	
Focal	15 (14)	9 (9)	2.7 (1.1 - 6.8)	
Diffuse	28 (26)	3 (3)	15.4 (4.0 - 59.7)	< 10^-4^
**Size**				
None	56 (53)	89 (88)	1	
< 3 cm	33 (31)	9 (9)	5.8 (2.5 - 13.8)	
≥3 cm	17 (16)	4 (4)	6.8 (2.1 - 22.2)	< 10^-4^

* Odds ratio with 95% confidence interval (95% CI) by logistic regression adjusted for matching variables (age, sex, hospital)

**p value from logistic regression

**Table 3:** Presence of peripheral neuropathies in cases and controls

	Cases n = 106 (%)	Controls n = 102 (%)	Odds Ratio* (95%CI)	*p *(chi^2^)
**Peripheral Neuropathies**				
none	85 (80)	94 (92)	1	0.009
axonal	7 (7)	6 (6)	1.3 (0.4 - 4.0)	
axonal with SCVs**	14 (13)	2 (2)	7.7 (1.6 - 36.6)	

**Radiculopathies**	24 (23)	14 (14)	1.8 (0.9 - 3.8)	0.09

*Odds ratio with 95% confidence interval (95% CI) by logistic regression adjusted for matching variables (age, sex, hospital)

** slowed conduction velocities

Radiculopathies were not significantly associated with the presence of SC-NFs (Table 3).

The joint analysis identified no interactions between variables associated with the presence of SC-NFs. A strong association was found between the presence of sciatic neurofibromas and that of axonal neuropathy with SCVs (aOR = 22.1; 95% CI, 4.2 to 115.3). In the bivariate analysis including axonal neuropathy with SCVs and sciatic neurofibromas, axonal neuropathy with SCVs was no longer associated with SC-NFs (aOR, 3.0; 95%CI, 0.6 to 15.1; *p *= 0.18). The association between axonal neuropathy with SCVs and SC-NFs persisted only in the subgroup with sciatic neurofibromas (aOR, 2.00; 95%CI, 1.01 to 4.12; vs. aOR, 0.69; 95%CI, 0.24 to 4.12 in the subgroup without sciatic neurofibromas). Thus, the association between SC-NF and axonal neuropathy with SCVs was due to the presence of internal sciatic neurofibromas.

**Figure 1:**
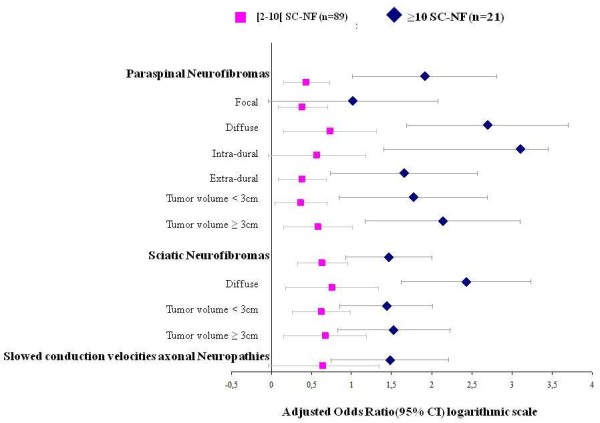
**Odds ratios (ORs) with their 95% confidence intervals for several types of internal neurofibromas and peripheral neuropathies in patients with two to nine subcutaneous neurofibromas (SC-NFs) or at least ten SC-NFs**. The ORs associated with each type of internal neurofibroma and peripheral neuropathy were estimated using multinomial logistic regression adjusted for the matching variables (age, sex, and centre), using the control group of patients with neurofibromatosis type 1 and no SC-NFs (n = 102) as the reference category. ORs were log-transformed.

In the multinomial logistic regression analysis, patients with at least ten SC-NFs had a significantly higher risk of paraspinal neurofibromas (aOR, 82; 95%CI, 10.4 to 647.9) compared to patients with two to nine SC-NFs (aOR, 2.7; 95%CI, 1.4 to 5.3) (Figure 1). This dose-effect relationship was observed for distribution, location, and size (Figure 1, Table 4). Similar dose-effect relationships were found for sciatic neurofibromas, particularly regarding distribution, location, size (Figure 1, Table 4). Finally, axonal neuropathies with SCVs were more common in patients with at least ten SC-NFs (aOR, 29.9; 95%CI, 5.5 to 162.3) than in patients with two to nine SC-NFs (aOR, 4.4; 95%CI, 0.9 to 22.0) (Figure 1).

**Table 4:** Association between subcutaneous neurofibromas (SC-NFs) and several types of internal neurofibromas and peripheral neurofibromas

	No SC-NFs (n = 102) Controls	2-9 SC-NFs (n = 85) OR** (95%CI)	≥10 SC-NFs (n = 21) OR** (95%CI)
**Paraspinal neurofibromas**	**1**	**2.7 (1.4 - 5.3)**	**82 (10.4 - 647.9)**
**Distribution**			
None	-	1	1
Focal	1	2.4 (1.2 - 5.0)	10.3 (0.9 - 120.0)
Diffuse	1	5.4 (1.4 - 20.4)	492.0 (48.4 - 5006.2)
**Location**			
None	-	1	1
Intradural	1	3.7 (0.9 - 15.0)	1270.0 (25.6 - 2849.8)
Extradural	1	2.4 (1.2 - 4.8)	45.0 (5.4 - 374.2)
**Size**			
None	-	1	1
< 3 cm	1	2.3 (1.1 - 4.9)	58.6 (6.9 - 494.0)
≥3 cm	1	3.8 (1.4 - 10.4)	136.7 (14.9 - 1253.9)

**Sciatic neurofibromas**	**1**	**4.3 (2.1 - 9.0)**	**29.1 (8.5 - 100.0)**
**Distribution**			
None	-	1	1
Focal	1	2.9 (1.2 - 7.0)	-
Diffuse	1	5.7 (1.5 - 21.7)	266.8 (41.6 - 1712.7)
**Size**			
None	-	1	1
< 3 cm	1	4.2 (1.8 - 9.8)	27.2 (7.2 - 103.2)
≥3 cm	1	4.7 (1.4 - 15.5)	33.4 (6.6 - 167.6)

Neuropathies with SCV***	**1**	**4.4 (0.9 - 22)**	**29.9 (5.5 - 162.3)**

*Reference category

**Odds ratio with 95% confidence interval (95%CI) by multinomial logistic regression adjusted for matching variables (age, sex, hospital), using the control group of patients with neurofibromatosis type 1 and no SC-NFs (n = 102) as the reference category.

*** Slowed conduction velocities

## Discussion

This study shows that sub-cutaneous neurofibromas were significantly associated with having paraspinal and sciatic neurofibromas detected by routine MRI. The strength of the association tended to be higher when the paraspinal and sciatic neurofibromas were diffuse, intradural, and at least 3 cm in diameter. Moreover, paraspinal and sciatic neurofibromas were significantly more common in patients having at least ten SC-NFs than in patients having two to nine SC-NFs. A dose-effect relationship was also found for distribution, location, and size of the internal neurofibromas. Finally, our data indicate that the strong association between SC-NFs and axonal neuropathy with SCVs is due to the association between SC-NFs and internal neurofibromas.

Neurofibromatosis-1 (NF1) is a common disease with an increased propensity for developing both benign and malignant tumors [10]. NF1 has been reported to be associated with a 15-year decrease in life expectancy [1]. One of the aims of the Réseau NF-France was to identify clinical predictive factors for mortality, in order to better adjust the follow up and propose new treatments for eligible patients. We previously demonstrated in two different NF1 populations, from France [4] and North America [5], that the presence of at least 2 subcutaneous neurofibromas was associated with a higher risk of death. The main causes of death were compression of neighboring organs due to internal neurofibromas, and malignant peripheral nerve-sheath tumours developing from preexisting internal neurofibromas.

The main concern in the long-term clinical management of adults with NF-1 is the identification of patients at high risk for MPNSTs developed from pre-existing internal neurofibromas. At present, no effective treatment is available for MPNST untreatable by surgery. New treatments are usually approved based on proof of efficacy in a controlled trial. When conducting controlled trials, an important consideration is patient selection based on severity of illness. Controlled trials in adults with NF-1 should target patients who have a high-risk phenotype defined by the presence of SC-NF. Patients with a high risk of internal neurofibromas must be therefore identified as potential new therapies are being developed. For instance, there are at least seven phase II/III trials in which the internal neurofibromas are being treated by targeted therapy such as ranibizumab, niltotinib... (clinicaltrials.gov). In addition, we believe that targeted therapy will be of greater efficacy on non-transformed internal neurofibromas. At present time, an annual careful clinical examination is recommended for all patients with NF1 in order to detect clinical symptoms associated with MPNSTs such as pain, neurological deficit and enlargement of a pre-existing peripheral nerve sheath tumour [11]. Routine MRI screening, which is highly reliable for detecting internal neurofibromas, is not recommended in patients without the alarming clinical signs mentioned above [12]. It is therefore critical to precisely clinically identify the patients with the maximum likelihood of having internal neurofibromas and to closely follow them by it iterating MRI. The first step is to assess recognizable clinical features as potential predictors of morbidity and mortality in order to further improve the clinical management of NF1. We recently developed a clinical score for predicting the presence of internal neurofibromas in adults with NF1. The NF1Score, computed via a linear combination of four variables (age ≤ 30 years, fewer than six café-au-lait spots, no cutaneous neurofibromas, and two or more SC-NFs), can be used to accurately predict the presence of internal neurofibromas in NF-1 patients and help target patients for now emerging clinical trial [6]. The high point scoring of the NF1Score is the presence of SC-NFs. SC-NFs were highly associated with having internal neurofibromas (OR, 4.7; 95%CI, 2.1-10.5) [6]. The second step, our present work, was to confirm and explain a detailed and unbiased characterisation of the links between internal neurofibromas and SC-NFs. To our knowledge, this was the first controlled study involving a detailed characterization of the relationship between SC-NFs, internal neurofibromas, and peripheral neuropathies in adults with NF-1.

The internal validity of this study depends on many factors, including the unbiased recruitment of cases and controls and the accuracy of the information obtained about the presence of internal neurofibromas. MRI, an extremely reliable method for detecting internal neurofibromas, was performed routinely in all our cases and controls. Assessment bias was minimized by having the MRI data reviewed by senior radiologist (PB) who was unaware of the clinical data. Assessment bias was minimized by having the electrophysiological data reviewed by senior physiologist (JPL) who was unaware of the MRI data. Clinical information regarding the neuropathy was not available. However, according to our experience the diffuse symmetric peripheral neuropathies related to NF-1 are asymptomatic or pauci-symptomatic with minor sensory manifestations [7].

As with all hospital-based case-control studies, selection bias cannot be excluded. However, the prevalence of the various clinical features in the cases were similar to those in the controls (except for the number of cutaneous neurofibromas) and to those reported previously in NF-1 patients, indicating that our study population was representative of NF-1 patients [13]. Regarding the number of cutaneous neurofibromas, the association linking the absence of cutaneous neurofibromas to the presence of SC-NF is in agreement with a previous study showing higher mortality among NF-1 patients who had no cutaneous neurofibromas [4].

In summary, this study shows a strong association between the presence of internal neurofibromas and the presence of SC-NF. The increased risk in NF-1 patients with subcutaneous neurofibromas (high risk phenotype) can be ascribed in part to the associations between SC-NFs and internal neurofibromas (prone to transformation to MPNSTs) and the presence of a neuropathy with SCVs. The prevalence of neuropathy with SCVs is particularly high in patients with at least ten SC-NFs. To better understand the patho-physiological processes in the high risk phenotype of NF-1 patients, the next step would be to perform genotype-phenotype correlation studies. A more severe clinical phenotype has been reported in NF1 patients carrying genomic microdeletions involving *NF1 *and surrounding gene compare to patients with mutations restricted to the *NF1 *genes. A recent study confirmed in a large cohort that NF1 microdeletion patients have a significantly higher incidence of learning disabilities and facial dysmorphism than patients with intragenic *NF1 *mutation [14]. However no association were found between SC-NFs and MPNSTs and NF1 microdeletion patients.

Further studies are therefore needed to find others genes beside *NF1 *implied in the internal NF development. One of the major aims of such studies would be to identify key targets for novel drugs' development.

## List of Abbreviations

aOR: adjusted Odds Ratio; CI: Confident Interval; MPNST: Malignant peripheral Nerve sheath tumor; MRI: magnetic resonance imaging; NF-1: Neurofibromatosis 1; SC-NF(s): Sub-Cutaneous Neurofibroma(s); SCVs: Slow Conduction Velocities; SD: standard deviation

## Competing interests

ES, SBG, LVA, SF, JPL, AD, PB, PC, SB, PW have non-financial interests that may be relevant to the submitted work.

## Authors' contributions

PW conceived and designed the study, and is guarantor for the study. SBG contributed to the concept and design, and supervised analysis and interpretation of the data. ES performed the data analyses and wrote the initial draft of the article, to which all the authors contributed. All authors had full access to the data in the study and can take responsibility for the integrity of the data and the accuracy of the data analysis. All authors have given final approval for the final version to be published. The NF-France network contributed to the patients' inclusion.
